# Elucidation of the substrate of tRNA-modifying enzymes MnmEG leads to *in vitro* reconstitution of an evolutionarily conserved uridine hypermodification

**DOI:** 10.1016/j.jbc.2022.102548

**Published:** 2022-09-28

**Authors:** Praneeth Bommisetti, Anthony Young, Vahe Bandarian

**Affiliations:** 1 Department of Chemistry, University of Utah, Salt Lake City, Utah, USA; 2 Soliome Inc, San Francisco, California, USA

**Keywords:** tRNA, RNA modifications, nucleic acid enzymology, tRNA methyltransferase, biochemistry, Cmnm, carboxymethylaminomethyl, EIC, extracted ion chromatograms, HRMS, high resolution mass spectrometry, LB, Lennox broth, mnm, methylaminomethyl, MTHFR, methylenetetrahydrofolate reductase, MWCO, molecular weight cut off, SHMT, serine hydroxymethyltransferase, SIM, selected ion monitoring, TCA, trichloroacetic acid, THF, tetrahydrofolate

## Abstract

The evolutionarily conserved bacterial proteins MnmE and MnmG collectively install a carboxymethylaminomethyl (cmnm) group at the fifth position of wobble uridines of several tRNA species. While the reaction catalyzed by MnmEG is one of the central steps in the biosynthesis of the methylaminomethyl (mnm) posttranscriptional tRNA modification, details of the reaction remain elusive. Glycine is known to be the source of the carboxy methylamino moiety of cmnm, and a tetrahydrofolate (THF) analog is thought to supply the one carbon that is appended to the fifth position of U. However, the nature of the folate analog remains unknown. This article reports the *in vitro* biochemical reconstitution of the MnmEG reaction. Using isotopically labeled methyl and methylene THF analogs, we demonstrate that methylene THF is the true substrate. We also show that reduced FAD is required for the reaction and that DTT can replace the NADH in its role as a reductant. We discuss the implications of these methylene-THF and reductant requirements on the mechanism of this key tRNA modification catalyzed by MnmEG.

Posttranscriptional modifications are present in all cellular RNA pools and many are conserved across all domains of life. To date, ∼140 posttranscriptional nucleoside modifications have been reported in the MODOMICS database, highlighting their importance and ubiquity (1). In particular, tRNA is one of the most extensively modified cellular RNA species, with the majority of modifications concentrated on the anticodon stem loop (2). Some modifications are introduced by a single step, while many are hypermodifications that are installed by complex multienzyme pathways (2, 3, 4, 5, 6, 7, 8, 9).

Modifications present on wobble uridines (position 34) of tRNA can be grouped based on the atom attached to the C-5 of the uracil (10) (Fig. 1). 5-Hydroxyuridine (xo^5^U) derivatives contain an oxygen atom bonded to position 5, whereas 5-methyluridine (xm^5^U) derivatives contain a methylene group attached to the uridine's position 5. Some of these wobble uridine modifications, such as the xo^5^U derivatives, expand anticodon recognition to three or four synonymous codons (10, 11, 12). By contrast, the xm^5^U derivatives are essential for the accurate translation of codons ending in A or G (NNA and NNG), while impeding the base pairing with codons ending in C and U (10). The importance of wobble uridine modifications is underscored by the fact that the loss of xm^5^U modifications in human mitochondria is seen in patients with mitochondrial encephalomyopathy, lactic acidosis, and stroke-like episodes (MELAS) and myoclonus epilepsy with ragged-red fibers (MERRF) (13, 14, 15, 16, 17, 18).

**Figure 1:**
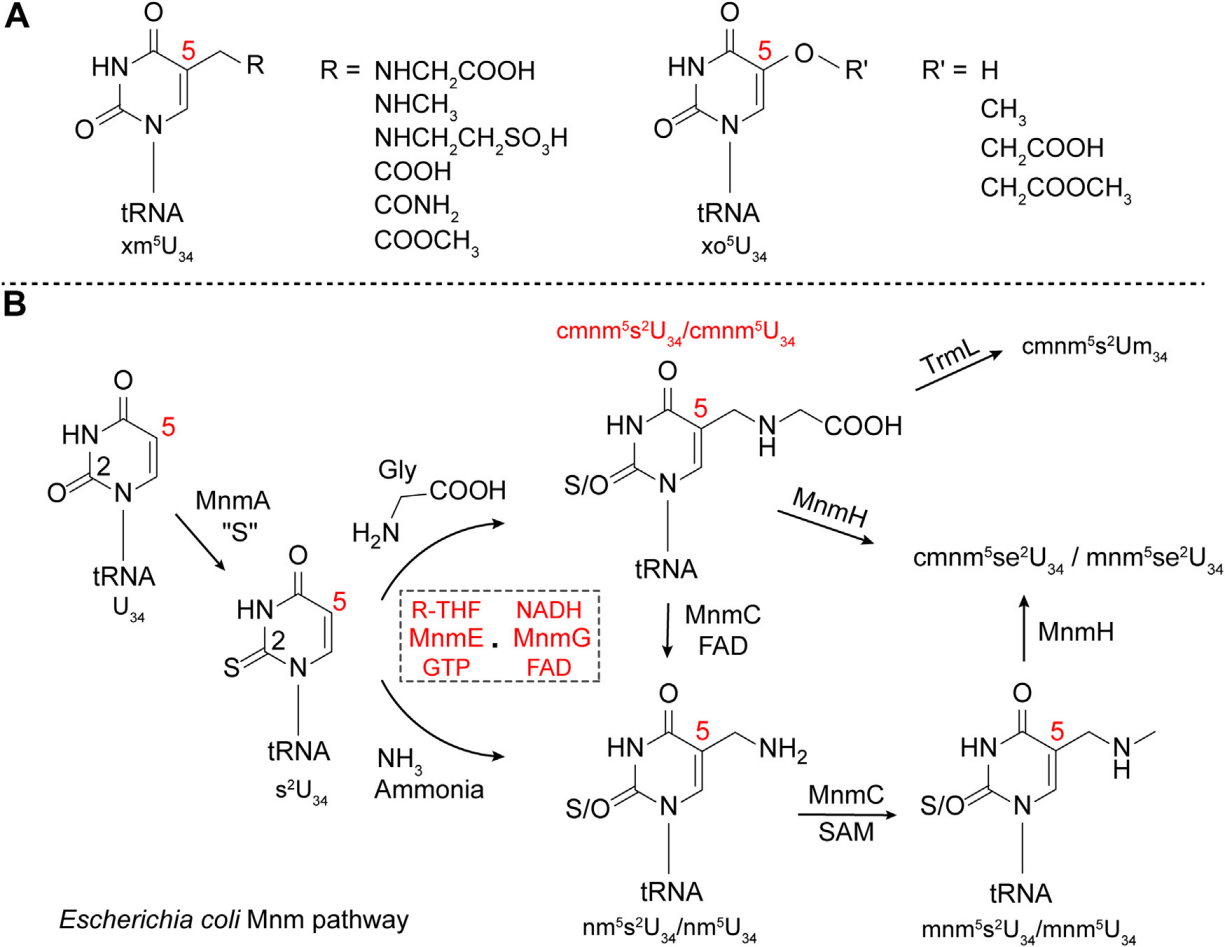
**Wobble uridine tRNA modifications.***A*, structures of xm^5^U and xo^5^U modifications where position 5 (denoted in *red*) of the base is modified. *B*, representative example of bacterial mnm pathway. The enzymatic reaction of interest in the current report is highlighted in *red*.

5-Carboxymethylaminomethyl (cmnm^5^) and 5-methylaminomethyl (mnm^5^) are two xm^5^U derivatives found at the wobble uridines of bacterial tRNA. The biogenesis of the mnm^5^ sidechain is initiated by MnmE and MnmG, which collectively convert U_34_ of tRNA^Arg^, tRNA^Gln^, tRNA^Glu^, tRNA^Gly^, tRNA^Leu^, and tRNA^Lys^ when present to their corresponding cmnm^5^U_34_ derivatives (1, 19, 20, 21, 22) (Fig. 1). The bifunctional enzyme MnmC catalyzes the flavin-dependent cleavage of the carboxymethyl moiety to form the aminomethyl (nm^5^), as well as S-adenosyl-L-methionine–dependent methylation to the mnm^5^ on tRNAs for Arg, Glu, Gly, and Lys (23, 24). In Leu tRNA, 2′-hydroxyl is further methylated by TrmL to convert to cmnm^5^U_m_ (1, 25). The thiolation at C-2 is catalyzed by MnmA and occurs independently of the modifications at C-5 on tRNAs for Gln, Glu, and Lys (26). When sufficient quantities of selenium are present in growth media, the SelU (or MnmH) inserts selenium at C-2 to form mnm^5^se^2^U in a fraction of tRNAs for Lys and Glu, completing the hypermodification pathway (27, 28, 29). The wobble xm^5^U modifications in mitochondrial tRNA are carried out by the homologs of MnmE and MnmG (such as MSS1 and MTO1 in *Saccharomyces cerevisiae* (30) or hGTPBP3 and hMTO1 in humans (31), see Fig. S1). However, taurine is appended instead of glycine to form a taurinomethyl (τm^5^) modified base (31). 5-Taurinomethyluridine (τm^5^U) and 5-taurinomethyl-2-thiouridine (τm^5^s^2^U) are examples of xm^5^U in human mitochondria (31).

While the role of the MnmEG complex (or its eukaryotic counterparts) in the cmnm modification is not disputed, several key aspects of this reaction remain unknown. First, MnmE has been shown to bind tetrahydrofolate (THF) and its derivatives such as methylTHF (CH_3_THF), methylene THF (CH_2_THF), and *N*^5^ or *N*^10^ – formylTHF (CHO-THF) with a similar affinity (20) (see Fig. S2 for structures). Interestingly, while THF does not have an available one-carbon unit at *N*^5^ to donate to the reaction, it has been shown that under some conditions, it can support the formation of the modified uridine base, suggesting that the enzyme likely copurifies with the true substrate for the reaction (20). Cocrystallization of 5-CHO-THF with MnmE has been taken as potential evidence that a formyl moiety is the source of the one-carbon (32). However, the enzyme is unlikely to be as promiscuous as these studies suggest. Indeed, CH_3_-, CH_2_-, and CHO-THF each present a one-carbon unit at a distinct oxidation state; moreover, THF does not carry a one-carbon unit.

The uncertainty likely results from the complex mixtures of THF species that often constitute commercially available THF derivatives, their well-known propensity to oxidation under aerobic conditions, and the ability of the enzyme to bind various THF analogs. As a counterpoint, studies examining mnm^5^ modification in strains that lack various genes in the biogenesis of the THF and its derivatives suggest that the one-carbon unit is derived from CH_2_THF (33, 34) (summarized in Fig. S3). However, there have been no studies that directly implicate the source of the one-carbon unit delivered by THF analog *in vitro* and in fact, as discussed previously, virtually all forms of THF have been proposed to support turnover. A second confounding issue is the role of a pyridine nucleotide in the reaction. NADH (but not NADPH) is required for the reaction (20). However, NADH was dispensable at higher FAD concentrations. This interdependence of NADH and FAD concentrations is also not understood. Despite all the studies, many questions remain about the exact nature of the mechanism of installation of the cmnm^5^ group by MnmEG.

Herein, we report *in vitro* biochemical reconstitution of the MnmEG reaction using well-defined isotopically enriched substrates. We show that while the protein copurifies with a variety of THF analogs when incubated with isotopically enriched CH_2_THF, isotope transfer from the THF analog to the product occurs. Moreover, we show that the reaction requires NADH but DTT can substitute in its absence. Spectrophotometric measurements with MnmEG clearly show that the endogenous FAD is reduced by NADH (or DTT). These observations lead to an outline of the mechanism of MnmEG that allow its classification as being analogous to FAD/folate-dependent C5 uracil methyl transferase proteins.

## Results

### MnmE and MnmG copurify with substrates and cofactors

**Figure 2:**
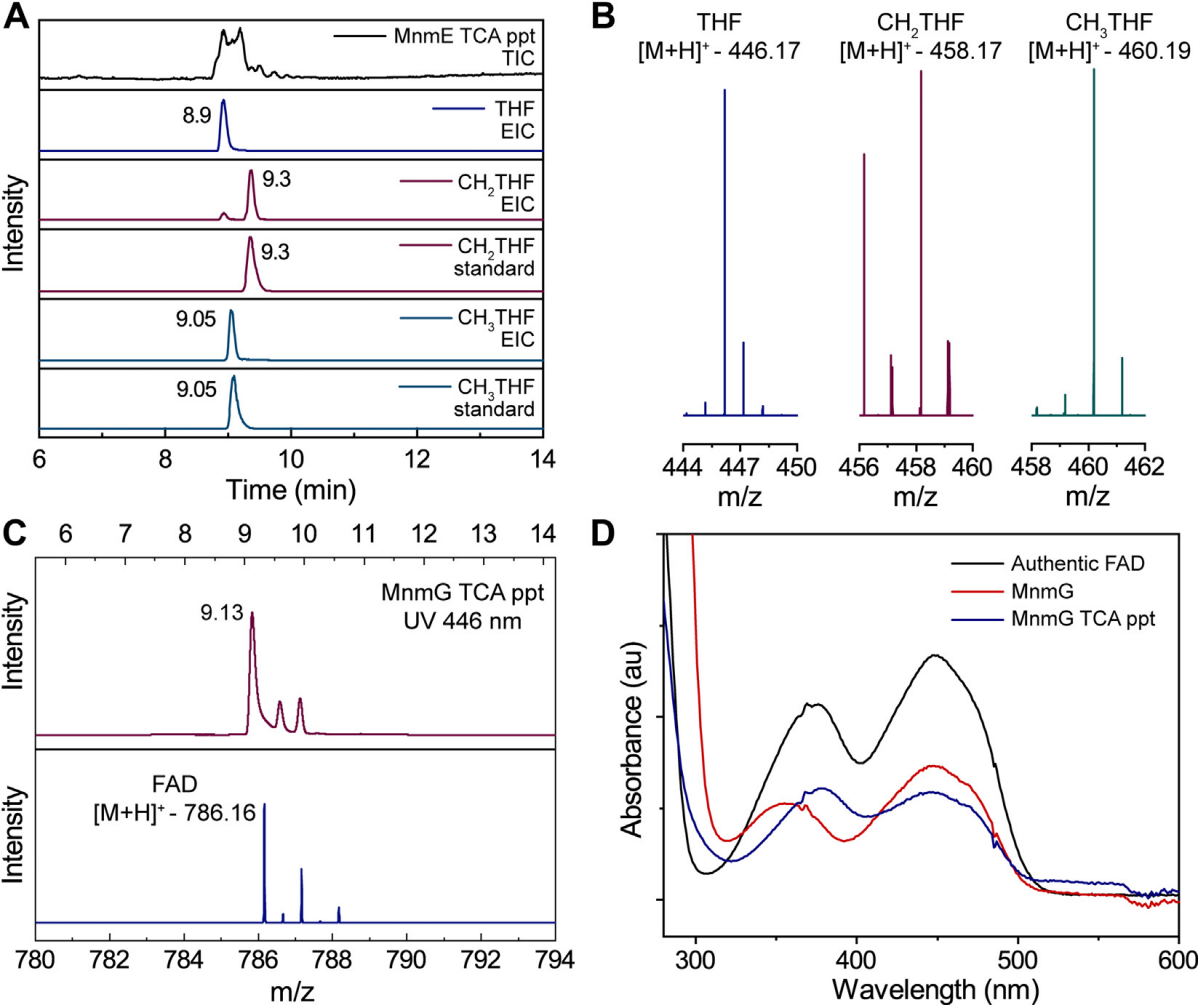
**Analysis of small molecules that copurify with MnmE and MnmG.***A*, the TIC of MnmE TCA precipitate (*black*) and EICs of THF (*m/z* – 446.17 ± 0.01, *blue*), CH_2_THF (*m/z* - 458.17 ± 0.01, *blue*), and CH_3_THF (*m/z* – 460.19 ± 0.01, *green*) from MnmE supernatant in comparison with commercially sourced CH_2_THF and CH_3_THF standards. *B*, representative mass spectra of the THF, CH_2_THF, and CH_3_THF observed in the MnmE supernatant sample *C*, representative LC trace at UV 446 nm from MnmG supernatant (*red*) and the observed mass spectrum for the FAD peak eluting at 9.13 min (*blue*). *D*, comparison of UV-vis spectra of intact MnmG (*red*), MnmG supernatant after TCA precipitation (*blue*), and commercially sourced FAD (*black*). The protein sample spectrum comparisons are made against ∼ 58 μM of authentic FAD. EIC, extracted ion chromatograms; LC, liquid chromatography; TCA, trichloroacetic acid; TIC, total ion chromatogram; THF, tetrahydrofolate.

MnmE and MnmG were expressed in *Escherichia coli* and purified to homogeneity *via* anion exchange and hydrophobic interaction chromatographic steps (see Fig. S4*A* for SDS-PAGE analysis). The GTPase MnmE has been shown to bind various THF derivatives with a similar affinity (20) and could copurify with the THF derivatives bound. Similarly, the flavoenzyme MnmG could copurify with the flavin cofactor. The protein solutions were dialyzed extensively before being concentrated to minimize the possibility of bound small molecules that could confound the results. However, to establish a baseline for all bound THF and flavin, the purified proteins were analyzed after denaturation with trichloroacetic acid (TCA). The analyses were carried out using HPLC–high resolution mass spectrometry (HRMS) analysis, and the resulting HPLC traces and MS data are shown in Figure 2.

The analysis of multiple MnmE preparations purified for this study shows that it indeed copurifies with THF, CH_2_THF, and CH_3_THF. The total ion chromatograms of supernatants from acid denatured MnmE depicted in Figure 2*A* (black trace) show that these molecules elute near one another under the employed liquid chromatography conditions. However, they can be clearly identified in the extracted ion chromatograms (EICs) and by comparison of their retention times to those of commercially sourced reagents. The mass spectra observed for the peaks at retention times 8.9, 9.1, and 9.3 min display *m/z* values of 446.17, 458.17, and 460.19, respectively, which are within 1 ppm of those expected for THF, CH_2_THF, and CH_3_THF, respectively (Fig. 2*B*). We also have mass spectrometric evidence for the presence of methenylTHF and traces of formylTHF (Fig. S5). Therefore, despite extensive efforts to remove any noncovalently associated molecules, the enzyme preparations carry measurable quantities of folates.

MnmG has previously been shown to be a flavoenzyme (35, 36). Purified recombinant *E. coli* MnmG is yellow, and the UV-visible spectrum of the protein (Fig. 2*D*) exhibits features at ∼350 and 450 nm, consistent with the presence of flavin. Upon treatment with TCA, the cofactor is released, and the spectrum of the cofactor in the solution is indistinguishable from free flavin (compare blue and black traces in Fig. 2*D*). To identify the nature of the bound flavin, supernatants from TCA-precipitated MnmG samples were analyzed by HPLC-MS. The MS of the species eluting at 9.13 min with the characteristic UV-visible features of flavin reveals an *m/z* value of 786.16, which is consistent with FAD (blue trace, Fig. 2*C*). Quantification of the FAD in the MnmG protein shows that it is not replete with flavin. In two different preparations of the protein used in these studies, the ratio of FAD to MnmG was found to be ∼1:5 to 1:6. Therefore, unless indicated otherwise, in the experiments described later, exogenous FAD was added to the assays to ensure maximal occupancy of the flavin-binding sites.

### *In vitro* reconstitution of MnmEG reaction

The collective actions of MnmEG are responsible for wobble uridine modification on tRNA in a reaction that utilizes glycine and a THF derivative as the cosubstrates (19). We sought to reconstitute the activity of MnmEG *in vitro* to probe the identity of the THF derivative. Since the flavin cofactor and the THF derivatives required for the MnmEG reaction are sensitive to air oxidation, all the assays were carried out in a glovebox under anoxic conditions. The hypomodified tRNA substrate for the reaction was obtained in the form of total tRNA from a *ΔmnmE* deletion strain of *E. coli* (Fig. S4*B*). In our initial assays, we supplied CH_3_THF or CH_2_THF *in trans*, along with FAD, NADH, GTP, and glycine. The reaction was allowed to proceed overnight (12–14 h) at 37 °C. The analysis for the presence of cmnm^5^s^2^U entailed extracting the tRNA from the reaction mixtures and digesting it to nucleosides using P1 nuclease, phosphodiesterase, and alkaline phosphatase and HPLC-HRMS analysis of the resulting nucleoside mixtures (37, 38).

**Figure 3:**
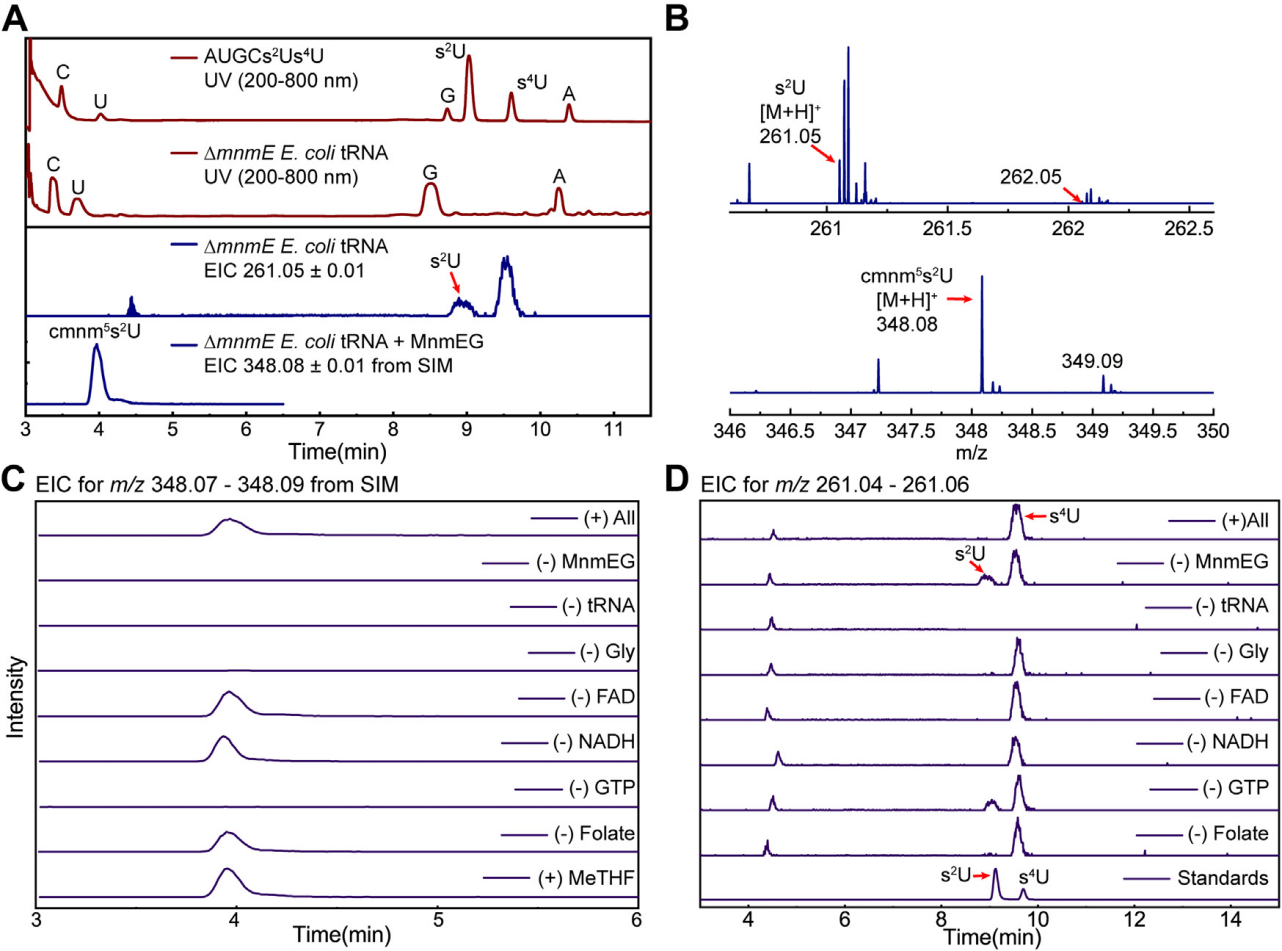
**HPLC-HRMS analysis of the tRNA nucleosides.***A*, representative PDA traces of nucleosides from either commercially sources or tRNA (*red*) and EICs of s^2^U (*m/z* - 261.05 ± 0.01) and cmnm^5^s^2^U (*m/z* – 348.08 ± 0.01) nucleosides from untreated and treated samples respectively (*blue*). *B*, representative mass spectra of the s^2^U and cmnm^5^s^2^U observed in untreated and treated samples, respectively, shown in (*A*) (*blue trace*). *C*, EICs of the cmnm^5^s^2^U (*m/z* – 348.08 ± 0.01) product from reaction samples obtained by omitting one reagent at a time. *D*, EICs of the s^2^U (*m/z* - 261.05 ± 0.01) nucleoside from reaction samples obtained by omitting one reagent at a time. The reaction samples are labeled in (*A*) and the omitted reagents are labeled for each of the traces in (*C*) and (*D*). Cmnm, carboxymethylaminomethyl; EIC, extracted ion chromatograms; HRMS, high resolution mass spectrometry; PDA, photodiode array.

The red traces in Figure 3*A* show UV chromatograms for a mixture of standard nucleosides (A, U, G, C, s^2^U, and s^4^U) and nucleosides obtained from digesting total tRNA from *ΔmnmE E. coli.* The nucleosides used as standards are well separated and allow for the assignment of peaks due to A, U, G, and C in the *E. coli* tRNA. The EIC trace at *m/z* = 261.05 for the digested *ΔmnmE E. coli* tRNA that is not treated with MnmEG shows peaks at 9 and 9.5 min, corresponding to the isobaric thiolated nucleosides s^2^U and s^4^U, respectively. In our experience working with thiolated nucleosides (s^2^U and s^4^U (38)), we observed that their MS signals are suppressed in the context of tRNA samples. To increase sensitivity, we employed selected ion monitoring (SIM) between 3 and 7 min to search for cmnm^5^s^2^U in the analysis of nucleosides from tRNA samples in reaction mixtures. The EIC corresponding to the mass of cmnm^5^s^2^U from a SIM experiment shows a peak at ∼4 min in the *ΔmnmE E. coli* tRNA sample treated with MnmEG. The observed mass spectra for s^2^U and cmnm^5^s^2^U from untreated and treated tRNA samples are shown in Figure 3*B*. As indicated previously, the thiolated nucleosides do not ionize well; therefore, their MS intensities are relatively weak. However, the peak for cmnm^5^s^2^U is clearly observed after treatment with MnmEG, and the observed *m/z* values for these nucleosides are within 3 ppm of theoretical values.

We next examined the role of various components of the assay mixture on the formation of cmnm^5^s^2^U (Fig. 3*C*) from s^2^U (Fig. 3*D*). The EIC traces show that in the presence of GTP, glycine, CH_2_THF, FAD, and NADH, the formation of cmnm^5^s^2^U is observed to be concomitant with the depletion of s^2^U. Removing MnmEG or the tRNA substrate abolishes the peak for cmnm^5^s^2^U. Interestingly, in the absence of glycine, s^2^U is depleted but no product forms. This observation may suggest a possible mechanistic paradigm for the MnmEG reaction (see Discussion). Curiously, however, we still observe the product even if FAD, NADH, or CH_2_THF are absent. These observations align with previous reports that product forms even in the presence of THF, which does not carry one carbon unit. Alternatively, we observe that CH_3_THF also supports product formation in a similar manner (Fig. S6). These seemingly contradictory observations on the role of THF are all likely because the protein copurifies with all the necessary components for at least one turnover. Since the enzymes are present in far excess over the substrate, which is likely a very small fraction of the total tRNA obtained from the deletion strain, it is not surprising that conversion is seen even in the absence of a THF substrate. However, we note that no product forms in the absence of GTP, pointing to the key role that the hydrolysis of the nucleotide plays in the reaction (39, 40, 41). These observations highlight and amplify ambiguities in the literature on the nature of the THF analog employed by MnmEG.

### Methylene source at the C5 of the uridine

**Figure 4:**
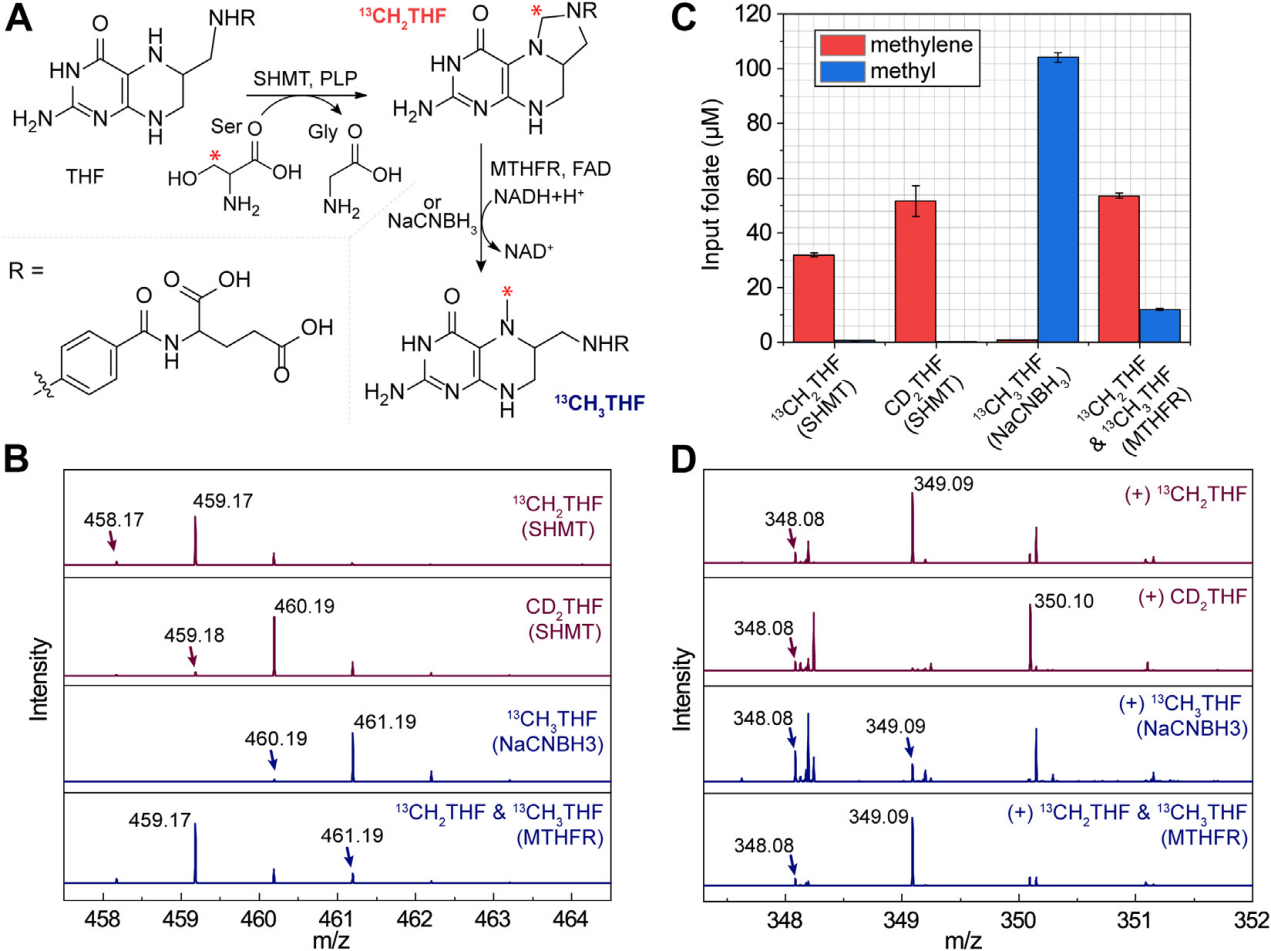
**Preparation and use with isotopically labeled THF derivatives in MnmEG reaction.***A*, schematic showing SHMT-based synthetic route to ^13^CH_2_THF and CD_2_THF, using β-^13^C-serine and β-CD_2_-serine, respectively. Reduction of ^13^CH_2_THF with NaCNBH_3_ or MTHFR/NADH was employed to prepare ^13^CH_3_THF. *B*, representative mass spectra of CH_2_THF and CH_3_THF HPLC peaks. *C*, quantification of isotopically enriched THF analogs obtained as shown in (*A*) by HPLC-HRMS. See experimental procedures for details on quantification. The error bars represent an average of two technical replicates for the folate solution used in the assays in panel (*D*). *D*, mass spectra of the cmnm^5^s^2^U nucleoside observed when various isotopically enriched THF analogs are used *in vitro* reconstitution of MnmEG activity. All of the samples contained GTP, NADH, and glycine. HRMS, high resolution mass spectrometry; SHMT, serine hydroxymethyltransferase; THF, tetrahydrofolate.

To directly probe the source of the carbon incorporated into the modified base, we synthesized THF derivatives where the appended one carbon unit was isotopically enriched. The isotopically enriched CH_2_THF isotopologs ^13^CH_2_THF and CD_2_THF were prepared by incubating serine hydroxymethyltransferase (SHMT) and THF with β-^13^C-serine or β-CD_2_-serine (33, 42) (Fig. 4*A*). The ^13^CH_3_THF isotopolog was prepared by two different routes from the ^13^CH_2_THF (described before) after filtering out the SHMT and reduction as follows. In the first, ^13^CH_2_THF was reduced enzymatically with methylenetetrahydrofolate reductase (MTHFR) and NADH (Fig. 4*A*). Alternatively, the filtrate containing ^13^CH_2_THF was treated with NaCNBH_3_ to obtain ^13^CH_3_THF (43) (Fig. 4*A*). The synthetic THF analogs were examined by LC-MS, and Figure 4*B* shows the MS corresponding to the chromatogram region corresponding to the analogs. While CH_2_THF has an *m/z* of 458.17, the synthetic ^13^CH2THF isotopolog has an *m/z* of 459.17, which is consistent with the presence of a ^13^C. The corresponding deuterated isotopolog (CD_2_THF) exhibits an *m/z* of 460.19, consistent with the presence of two deuterium atoms. The reduction of ^13^CH_2_THF with NaCNBH_3_ cleanly yields the corresponding reduced ^13^CH_3_THF (*m/z* of 461.19), with only a trace amount of ^13^CH_2_THF remaining. By contrast, reduction with MTHFR/NADH yields a mixture of ^13^CH_2_THF (*m/z* = 459.17) and ^13^CH_3_THF (*m/z* = 461.19).

The THF analogs were quantitated using the HPLC-HRMS analysis. The CH_2_THF isotopologs prepared by SHMT contain mostly (98%–99%) the desired CH_2_THF derivative (Fig. 4*C*). However, the subsequent reduction to obtain CH_3_THF yielded varying amounts of ^13^CH_3_THF and ^13^CH_2_THF, depending on the nature of the reducing equivalent (see Fig. 4*C*). While reduction with NaCNBH_3_ converts the ^13^CH_2_THF to ^13^CH_3_THF quantitatively (∼99.5% conversion), reduction with MTHFR and NADH yields a mixture of labeled ^13^CH_2_THF and ^13^CH_3_THF in a ratio of 82:12. In all cases, the reaction mixtures were filtered through a 10 KDa molecular weight cut off (MWCO) filter to remove the protein, and the resulting THF analogs were directly used in reactions with MnmEG without any further purification.

The mass spectra cmnm^5^s^2^U nucleoside formed upon incubation of the CH_2_THF isotopologs synthesized as described previously with MnmEG are shown in Figure 4*D*. As shown previously (see Fig. 2*B*), incubation with unlabeled folate yields a nucleoside product with an *m/z* of 348.08. By contrast, when ^13^CH_2_THF methylene is utilized, a nucleoside product with a +1 amu shift in mass forms (*m/z* = 349.09) (Fig. 4*D*). By analogy, using CD_2_THF led to a product with a +2 amu shift in mass to exhibit an *m/z* of 350.10, consistent with the incorporation of two deuteriums into the nucleoside product. A small amount of unlabeled product (*m/z* 348.08) is also observed, presumably from endogenously bound CH_2_THF or small amounts of unenriched CH_2_THF present in the input folate sample. To rule out CH_3_THF, however, we also carried out the same experiments with ^13^CH_3_THF prepared by NaCNBH_3_ reduction, where an unlabeled product with *m/z* of 348.08 is observed (compare Figs. 4*D* and 2*B*). We note that the minor enrichment of the 349.09 peaks can be attributed to the ∼0.5% amounts of CH_2_THF that remains after the reduction. When the ^13^CH_3_THF prepared by MTHFR/NADH is used, most of the product obtained is enriched (*m/z* = 349.09). This results from the significant quantities of ^13^CH_2_THF that remain after the enzymatic treatment. Collectively, these data unambiguously establish that the source of the carbon atom in cmnm^5^s^2^U is CH_2_THF.

### Formation of cmnm^5^s^2^U by MnmEG requires reducing equivalents

MnmG is a flavoenzyme, and it has been presumed that the formation of cmnm^5^s^2^U requires reducing equivalents (36, 44). However, the role of the reductant in tRNA modification reaction is not known (19, 20). In the studies shown in Figure 3, we observed, curiously, that eliminating exogenously supplied FAD and NADH from the reaction mixtures does not eliminate the formation of cmnm^5^s^2^U. The result with FAD can be justified because the enzyme already has some bound FAD, and one can rationalize that it is present in sufficient quantities for a significant fraction of MnmG to be catalytically active. Therefore, we sought to probe the requirement more directly for a reductant.

**Figure 5:**
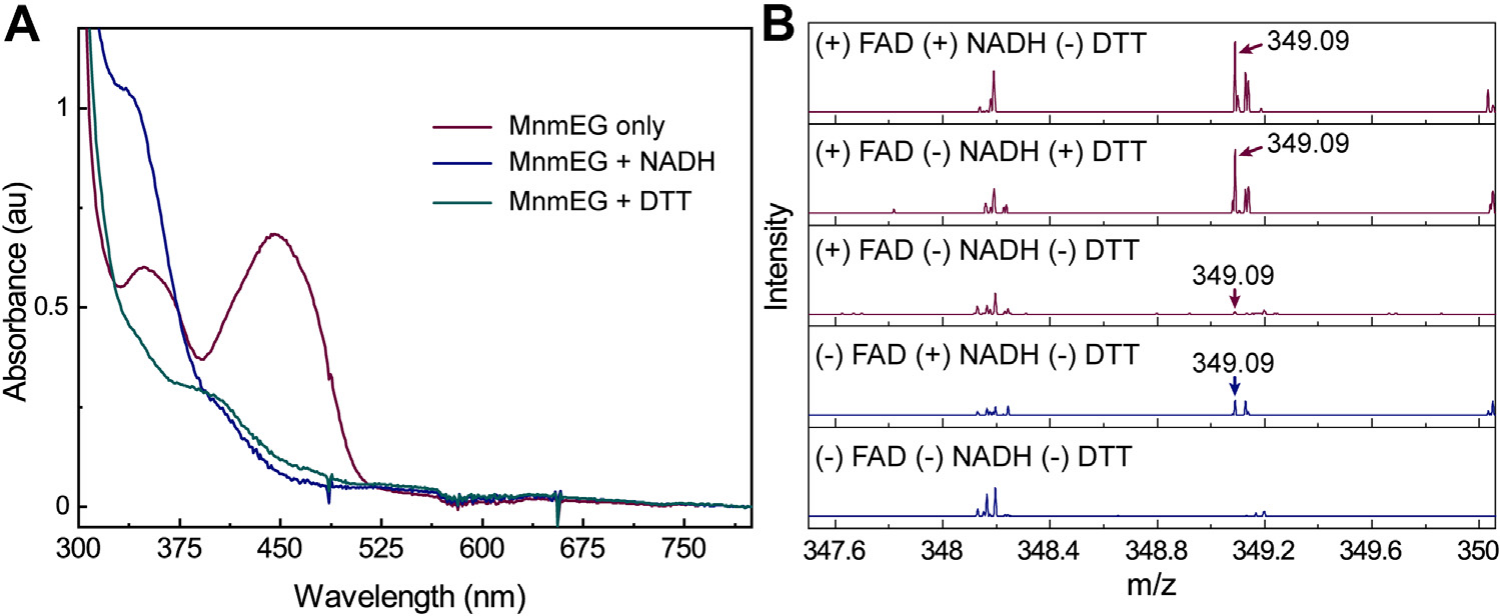
**Role of reductant in the MnmEG reaction.***A*, UV-visible spectrum showing the loss of characteristic 450 nm absorbance by endogenously bound FAD in DTT-free MnmEG complex after incubation with exogenously supplied reducing agent under anaerobic conditions. On the contrary, endogenously bound FAD remains oxidized in the MnmEG only containing sample (*green trace*). Approximately 200 μM protein solutions were incubated with either 200 μM NADH or 1 mM DTT. *B*, the mass spectra of cmnm^5^s^2^U nucleoside showing product formation or absence when either FAD, NADH, and DTT are removed individually or in combination. The included or removed reagents are labeled for each trace. The *y*-axis is scaled similarly for all the spectra to facilitate direct comparison.

Our preparations of MnmEG and all studies on the proteins that have been published to date are carried out in the presence of DTT (20, 32, 44). Therefore, the NADH paradox may be because there is enough DTT in the reaction mixtures to substitute when NADH is absent. Therefore, to probe the role of DTT, it was omitted from purification buffers and NADH or DTT were supplied exogenously. The UV-visible spectra depicted in Figure 5*A* show that when DTT-free MnmEG is incubated with NADH under anaerobic conditions, the endogenous flavin turns colorless within 20 min of incubation, as evidenced by the bleaching of the 450 nm peak (blue trace). A similar observation is also made within 90 min when DTT-free MnmEG proteins are incubated with exogenously supplied DTT (green trace). By contrast, when a reductant is absent, the bound flavin remains oxidized (red trace). Not surprisingly, we also observed free flavin reduction by DTT in the solution overnight under anaerobic conditions (Fig. S7). The exact nature of bound flavin reduction by DTT is not further explored. However, these observations clearly show that the flavin cofactor is likely reduced under the conditions that the assays are generally conducted with MnmEG.

Next, we examined the formation of cmnm^5^s^2^U with MnmEG purified using DTT-free buffers in the presence and absence of exogenously supplied FAD and reducing equivalents in the form of DTT or NADH. α-^13^C-Glycine was used in these assays to show that any observed product forms in the reaction unambiguously. As shown in Figure 5*B*, when MnmEG is incubated with GTP, α-^13^C-glycine, tRNA, CH_2_THF, and FAD in the presence of NADH, a peak at *m/z* of 349.09 is observed, consistent with the formation of ^13^C-labeled cmnm^5^s^2^U, which has expected *m/z* of 349.09. Turnover is also observed in the absence of NADH but in the presence of DTT. However, in the absence of both NADH and DTT, only a trace amount of ^13^C-labeled cmnm^5^s^2^U is observed. We could not eliminate the possibility that small amounts of the purified protein are reduced *in vivo* and remain reduced during the purification or that a small fraction is photo reduced during the assays. Further, the data show that the exogenous FAD is dispensable for the formation of cmnm^5^s^2^U, as the modification occurs if NADH is present. Finally, no product forms without NADH, DTT, and exogenously supplied FAD. These observations clearly show that the formation of cmnm^5^s^2^U requires the presence of a reducing equivalent, provided *in vivo* by NADH, but can be bypassed *in vitro by* DTT. Moreover, exogenous FAD is not necessary, and the bound MnmG is sufficient to support the reaction.

## Discussion

The presence of cmnm^5^s^2^U and mnm^5^s^2^U hypermodified bases in *E. coli* tRNA was known (45, 46) long before their biosynthetic enzymes were identified and a role for them in base modification was established. For example, *gidA*, later renamed *mnmG*, was discovered in the context of encoding a protein that led to a glucose-inhibited cell division phenotype in bacteria (47). However, a study examining the frameshifting of GA repeats in *E. coli* using a β-galactosidase reporter assay led to the isolation of variants lacking *mnmE* or *mnmG*, establishing a potential link between these proteins and translation (21). A similar inference was also made in another study examining *E. coli* variants deficient in the synthesis of mnm^5^s^2^U, which led to the recognition that the strains lack either *mnmE* or *mnmG* (22). Contemporaneously, genes encoding for MnmA and MnmC were already known and characterized to some extent, leading to the hypothesis that MnmE and MnmG, along with MnmA, collectively constitute the pathway to cmnm^5^s^2^U (21).

In the early 2000s, biochemical and structural studies on MnmE and MnmG established them as a GTPase and flavoenzyme, respectively (32, 35, 36, 39, 41, 44, 48). However, the *in vitro* reconstitution of the modification has proven challenging. MnmE, for example, can bind virtually all proffered THF analogs, including THF that carries no one carbon unit (20). Though levels of hypomodified bases that accumulate in strains lacking folate pathway enzymes suggest that CH_2_THF is the substrate (20, 33, 34), this has never been demonstrated directly. Moreover, the molecular imperatives for cofactors, FAD and NADH, or the precise role of GTP and the exact nature of the reaction mechanism remained elusive. It has, for example, been challenging to understand why modification would sometimes require reductant (in the presence of low FAD concentrations) and at other times proceeded without the need for reductant (at high FAD concentrations) (20).

The results in this article resolve these long-standing questions on substrate and reductant requirements of MnmEG. The uncertainties in the literature likely result from the propensity of MnmE to copurify with a variety of THF analogs, including CH_2_THF. The reductant requirement ambiguities result from variable quantities of DTT present in all previously purified MnmEG proteins. Using isotopically enriched THF analogs, we show that CH_2_THF is the correct substrate for the enzyme reaction. Moreover, we also demonstrate that the reaction requires NADH, though DTT can substitute in its absence. These results suggest that a reduced flavin is the starting point of the reaction that mobilizes the methylene carbon of the CH_2_THF, appending it to the C-5 position of the uracil base in the pathway to cmnm.

**Figure 6:**
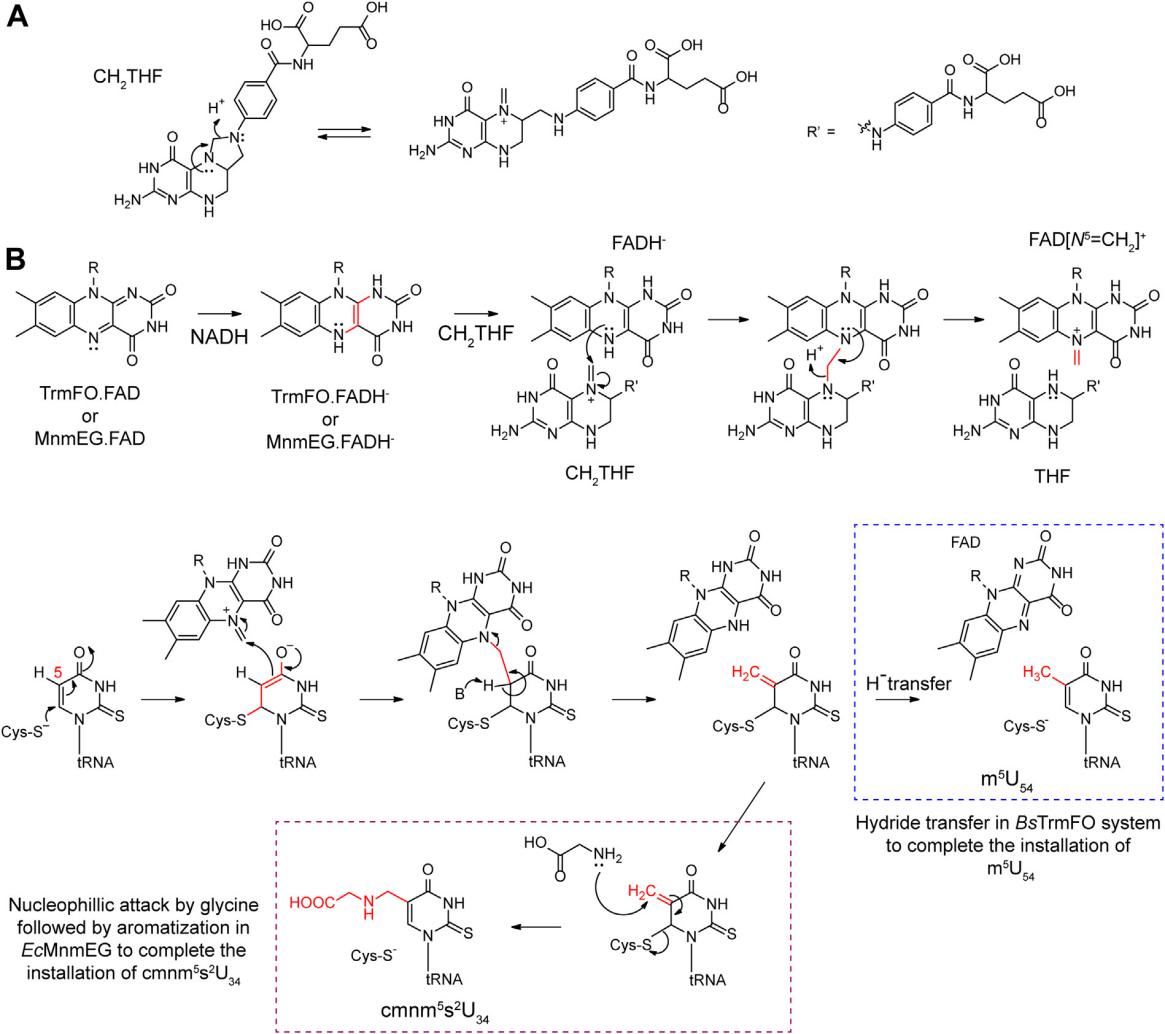
**Schematic comparing the mechanisms of *Bacillus subtilis (Bs)* TrmFO and *E. coli (Ec)* MnmG.***A*, resonance structures of the CH_2_THF. *B*, proposed mechanism of TrmFO in the literature where the reduced flavin accepts CH_2_ group from the CH_2_THF and transfers it to the C-5 of the uracil. A similar mechanistic pathway could be proposed for the *Ec*MnmEG reaction. In TrmFO, a hydride transfer from the FADH^−^ leads to formation of the m^5^U (*blue dashed line* box), whereas in MnmEG, nucleophilic attack by the glycine leads to the final product (*red dashed line* box). Both proteins utilize NADH to generate a reduced flavin cofactor and CH_2_THF as the carbon source at the C5 of the uracil. THF, tetrahydrofolate.

The requirement for CH_2_THF and NADH in modification of the C-5 position of uracil by MnmEG in a FAD-dependent manner is reminiscent of folate/FAD-dependent TrmFO, which catalyzes a similar reaction and installs a methyl group onto C5 of the uridine present at position 54 of tRNAs in Gram-positive bacteria (49). Unlike in MnmEG, where each of the MnmE and MnmG binds to CH_2_THF and FAD, respectively, TrmFO binds to both CH_2_THF and the flavin cofactor (50). CH_2_THF can exist as a iminium tautomer in solution as shown in Figure 6*A*. The proposed mechanism for the TrmFO involves the formation of the iminium species by covalent transfer of the methylene group from the CH_2_THF to the reduced flavin cofactor (50, 51, 52, 53) (Fig. 6*B*). Further, the uracil on the tRNA is activated by an active site Cys residue allowing it to attack the FAD[*N*^5^ = CH_2_]^+^, forming a crosslinked species, which is resolved by deprotonation at C-5. Hydride transfer from the reduced flavin cofactor to olefin leads to m^5^U (50, 51, 52, 53).

TrmFO and MnmG both belong to the Gid class of proteins. MnmG clusters with the GidA_L_ subclass in which the representative proteins are ∼600 aa long. By contrast, TrmFO is in the GidAs subclass, which is generally shorter (∼450 aa) (54). By analogy to TrmFO, a mechanistic proposal for the MnmEG reaction based on the substrate's identity, reducing equivalent requirement, and conserving Cys residue is shown in Figure 6*B*. We propose that MnmEG catalyzes cmnm^5^ installation by employing a reduced flavin as a way station for the one-carbon from CH_2_THF to the base. However, the final step in the MnmEG reaction entails trapping the olefin by the amino group of glycine in contrast to hydride transfer from reduced flavin in the TrmFO reaction (55). Further, in mammalian MnmEG homologs, taurine substitutes for glycine (31).

Besides tRNA modification, MnmG is also involved in other regulatory pathways. The gene encoding for MnmG is also potentially involved in regulating the pathogenicity and virulence of certain microbes. Specifically, a *gidA* mutant of the pathogen *Salmonella enterica* serovar Typhimurium displayed high attenuation in a *Salmonella* infection mouse model (56, 57). Additionally, *Salmonella gidA*^*−*^ strain exhibits downregulation of important virulence factors and alteration in several stress response genes for cold shock, heat shock, and nutrition deprivation compared to the WT (56, 57). To amplify their importance, the proteins MnmE and MnmG have been included in the list of a minimal set of proteins required to have a functional translational apparatus in bacteria (58, 59). The discovery in this article of the substrate and reductant requirements of MnmEG pave the way for detailed mechanistic studies of this RNA modification system as a potential target of therapeutic interventions.

## Experimental procedures

### Cloning of the *mnmE, mnmG, glyA, and metF*

The *mnmE* and *mnmG* gene nucleotide sequences were PCR amplified from the *ΔqueA E. coli* (from Keio collection (60), *E. coli* K-12 substrain) genomic DNA using Phusion High-Fidelity DNA polymerase (New England Biolabs) following the manufacturer's instructions. An annealing temperature of 68 °C was used based on the primer sequences designed for the PCR amplification. The forward and reverse primers used for *mnmE* PCR amplification were 5′- AAAAAAAA*CATATG*AGCGATAATGACACTATCG-3′ and 5′- AAAAAAAA*AAGCTT*TTACTTACCAATACAGAAGCTGGAG-3′, respectively, with italicized sequence representing *Nde*I and *Hind*III restriction cut sites. The PCR products were digested with *Nde*l and *Hind*III and ligated with T4 DNA ligase into similarly digested and alkaline phosphatase treated pET29a vector to obtain pAY756 used in the expression of MnmE in *E. coli.* The plasmid to express MnmG was prepared the same as aforementioned, except the forward and reverse primers were 5′-AAAAAAAA*CATATG*TTTTATCCGGATCCTTTTGACGTCATCATC-3′ and 5′-AAAAAAAA*CTCGAG*TTATGCGCTACGACGCAGCATACCC-3′, respectively, with italicized sequence representing *Nde*I and *Xho*I restriction cut sites. The resulting expression plasmid was named pAY760 and used to prepare MnmG.

The *glyA* and *metF* genes for SHMT and MTHFR enzymes were cloned from the *Δtgt E. coli* (from Keio collection, *E. coli* K-12 substrain) genomic DNA using Phusion High-Fidelity DNA polymerase (New England Biolabs) following the manufacturer's instructions. An annealing temperature of 66 °C was used for both the PCR amplifications. The forward and reverse primers used for *glyA* were 5′- AGAACCTGTACTTCCAGGGC*CATATG*TTAAAGCGTGAAATGAACATTGC-3′ and 5′- GGTGCTCGAGTGCGGCCGC*AAGCTT*TTATGCGTAAACCGGGTAACG-3′, respectively, with italicized sequences representing the *Nde*I and *Hind*III restriction cut sites. The forward and reverse primers used for *metF* were 5′-AGAACCTGTACTTCCAGGGC*CATATG*AGCTTTTTTCACGCCAGC and 5′-GGTGCTCGAGTGCGGCCGC*AAGCTT*TTATAAACCAGGTCGAACCCCC-3′, respectively, with italicized sequences similarly representing the *Nde*I and *Hind*III restriction cut sites. The PCR products were incorporated into the pET28t vector in a single step using the NEBuilder HiFi DNA assembly cloning kit following the manufacturer's instructions. The pET28t plasmid contains an N-terminal His-tag followed by a Tobacco Etch Virus nuclear-inclusion-a endopeptidase (TEV) cleavage site fused to the protein (37, 61).

### Production of MnmE, MnmG, SHMT, and MTHFR proteins

MnmE, MnmG, SHMT, and MTHFR plasmids are all transformed into *E. coli* BL21-DE3 phage T1 resistant electrocompetent *E. coli* cells (New England Biolabs) and inoculated onto Lennox broth (LB) agar plates containing kanamycin (34 μg/ml). The plates were incubated at 37 °C overnight to allow for the growth of the colonies. A single colony was picked from each plate and inoculated into ∼120 to 130 ml LB media and subsequently grown at 37 °C overnight by shaking at 200 rpm.

Large scale expression was carried out in 6 to 12 2.8 l Fernbach flasks containing 1 l of LB media. An aliquot of the overnight cultured bacteria (10 ml) was added to each flask containing media and kanamycin (34 μg/ml). The cells were grown at 37 °C by shaking at 175 to 200 rpm until the *A*_600_ of the culture reached ∼0.5 to 0.6, at which point the cells were induced by the addition of IPTG to a final concentration of 100 μM. Flasks containing the bacteria expressing the MnmG were further supplemented with solid riboflavin (∼50 mg/l) after the IPTG induction to support the overexpression of the flavoprotein. The induced cells were shaken at 150 to 175 rpm overnight (∼12–16 h) at 37 °C (for MnmE and MnmG) or 18 °C (for SHMT or MTHFR). The cells were harvested by centrifugation at 5000*g* for 10 min, flash frozen in liquid nitrogen, and stored at –80 °C until further use.

### Purification of the proteins MnmE and MnmG

The cells obtained from 12 l of media with cells expressing either MnmE or MnmG were resuspended in ∼150 ml of 20 mM Tris–HCl pH 8.0, 2 mM DTT, and 1 mM PMSF to inhibit proteases. The resuspended cells were lysed in the cold room at 4 °C or on ice by a sonicator with 50% amplitude, 15 s pulses, and 30 s rest to allow temperature re-equilibration. The cell lysate was clarified by centrifugation at 18,000*g* for 60 min at 4 °C and loaded onto a 40 ml Q Sepharose FF column (2.6 cm (D) × 14.5 cm (H), resin from GE healthcare) pre-equilibrated in buffer containing 20 mM Tris–HCl pH 8.0 and 2 mM DTT. After loading the lysate, the column was washed with the same buffer until the absorbance returned to the baseline, at which point the protein was eluted by a linear gradient from 0% to 100% 1 M KCl in a buffer containing 20 mM Tris–HCl pH 8.0 and 2 mM DTT over 5 to 10 column volumes. Fractions containing the protein of interest were identified by SDS-PAGE analysis, pooled, and then purified further as described later.

To the protein eluted from the anion exchange column, solid ammonium sulfate was added directly while stirring in the cold room at 4 °C to a final concentration of 1 M. The small amount of protein precipitated during this step was removed by centrifugation at 18,000*g* for 20 min at 4 °C. The clarified protein solution was loaded onto a butyl Sepharose FF column (2.6 (D) × 14.5 (H) cm, resin from GE healthcare) pre-equilibrated in the loading buffer containing 20 mM Tris–HCl pH 8.0, 1 M (NH_4_)_2_SO_4_, and 2 mM DTT. After loading, the column was washed until the absorbance returned to the baseline. At this point, the protein was eluted with a gradient of the loading buffer without ammonium sulfate. The protein of interest was identified by SDS-PAGE analysis. The pooled fractions were dialyzed against 4 l of buffer containing 20 mM Tris–HCl pH 8.0, 2 mM DTT, and 5 % glycerol. The dialysis buffer was changed thrice, and the dialyzed protein was then concentrated using Amicon with an MWCO filter of (30 kDa). DTT-free MnmE and MnmG were prepared by purifying the proteins similarly as described before, although DTT was omitted from the purification buffers except for the buffer in which cells were resuspended and lysed. A representative SDS-PAGE analysis of MnmE and MnmG purified proteins is shown in Fig. S4*A*. The concentrated proteins were flash-frozen in aliquots, and the concentration was determined using the Bradford assay with bovine serum albumin as standard.

### Purification of the proteins SHMT and MTHFR

The cells obtained from the overnight growth (for SHMT and MTHFR) were resuspended in ∼50 to 100 ml of buffer containing 50 mM potassium phosphate (KPi) pH 7.4, 500 mM KCl, 50 mM imidazole, and 1 mM PMSF. The resuspended cells were lysed using a sonicator with 15 s bursts at 50% followed by 30 s rest for temperature re-equilibration. The cell lysate was clarified by centrifugation at 18,000*g* for 60 min at 4 °C and loaded onto two serially connected 5 ml HisTrap FF (Cytiva) columns charged with Ni^2+^ and pre-equilibrated with a buffer containing 50 mM KPi pH 7.4, 500 mM KCl, and 50 mM imidazole. The column was washed with the loading buffer until the absorbance reached baseline. The proteins were eluted with a gradient to 500 mM imidazole in the loading buffer over 60 ml. The desired protein fractions were pooled and dialyzed against a buffer containing 50 mM Hepes-NaOH pH 8.0, 150 mM KCl, and 2 mM DTT. After SDS-PAGE analysis, SHMT seemed sufficiently pure and therefore was concentrated, frozen, and stored until use. The MTHFR from the first step was further purified by treating it with TEV protease (37, 61) to remove the N-term His_6_-tag on the protein. The reaction was carried out in the dialysis bag overnight in the cold room at 4 °C. The resultant solution was loaded onto two serially connected 5 ml HisTrap FF (Cytiva) columns charged with Ni^2+^ and pre-equilibrated with a buffer containing 50 mM KPi pH 7.4, 500 mM KCl, and 50 mM imidazole. The fractions of interest were identified by SDS-PAGE analysis, pooled, and dialyzed overnight against a buffer containing 50 mM Hepes-NaOH pH 8.0, 150 mM KCl, and 2 mM DTT. The dialyzed protein was then concentrated, flash frozen, and stored at −80 °C until further use. The concentrations of the proteins were determined using the Bradford assay with bovine serum albumin as standard. A representative SDS-PAGE analysis of SHMT and MTHFR preparations is shown in Fig. S4*A*.

### Purification of total tRNA from *ΔmnmE E. coli*

The hypomodified tRNA substrate containing s^2^U at the wobble uridines was obtained from the *E. coli ΔmnmE* deletion strain (from the Keio Collection (60)). LB culture media containing 34 μg/ml kanamycin (∼120–130 ml) were inoculated with the strain and grown overnight (12–16 h) at 37 °C and shaking speed of 200 rpm. An aliquot (10 ml) of the overnight culture was introduced into each of the six 2.8 l Fernbach flasks containing 1 l of LB media with 34 μg/ml kanamycin. The cultures were allowed to grow overnight at 37 °C by shaking at 200 rpm. The cells were pelleted by centrifugation at 5000*g* for 10 min at 4 °C. The resultant cells were flash frozen until further use. RNase-free water was used where applicable to avoid tRNA degradation during the preparation. The RNase-free water is prepared by treating the Millipore filtered water with 1 ml diethylpyrocarbonate per liter of the water to inactivate the RNases, followed by sterilization by autoclave to hydrolyze the remaining diethylpyrocarbonate.

The RNA extraction is carried out essentially as described before but with modifications to the purification of the extracted RNA (37). The cells were resuspended in a buffer containing 10 mM Tris–HCl pH 8.0, 10 mM MgCl_2_, and 150 mM NaCl (∼1 ml/1 mg of wet cell paste) by stirring in the cold room at 4 °C for 60 min. Next, saturated phenol (pH 4.3, 1 ml/1 mg of wet cell paste) was added to the resuspended cells and stirred for 60 min in the cold room at 4 °C. The resulting suspension was poured into RNase-free 50 ml conical tubes and centrifuged at 10,000*g* for 30 min at 4 °C. The upper aqueous layer was then transferred to fresh 50 ml conical tubes, and saturated phenol (pH 4.3, 1 ml/mg of starting wet cell paste) was added and mixed by inversion. The resulting suspension was centrifuged at 10,000*g* for an additional 30 min at 4 °C. The resulting upper aqueous layer was removed and mixed with 0.1 volumes of 3 M sodium acetate (pH 5.2) and three volumes of ice-cold ethanol. The resulting solution was incubated at –20 °C overnight to precipitate RNA. The RNA was pelleted by centrifugation at 15,000*g* for 30 min at 4 °C, the supernatant was discarded, and the pellet was resuspended in 1 M NaCl solution by vigorous vortex mixing. The undissolved precipitate was removed by centrifugation at 7000*g* for 30 min at 4 °C. The supernatant was collected and ethanol precipitated as described previously. Further purification of the tRNA was carried out on refrigerated AKTA fast protein liquid chromatography prime setup using an anion exchange column. The pump, super loop, and the lines were thoroughly washed with RNase-free water before purifying the RNA.

The pellet from the second ethanol precipitation was dissolved in 40 to 50 ml of a buffer containing 20 mM sodium acetate (NaOAc) pH 5.2 and loaded onto a 50 ml DEAE Sepharose FF column (resin from Cytiva) pre-equilibrated with the same buffer. The column was then washed with a loading buffer containing 0.34 M NaCl until the absorbance returned to the baseline. At this point, the RNA was eluted with a gradient from 0.34 M to 0.52 M NaCl in a total of 875 ml. The fractions were analyzed *via* 10% denaturing PAGE analysis (TBE buffer system, see Fig. S4*B* for a representative RNA elution profile on the DEAE Sepharose column). The tRNA fractions were pooled and ethanol precipitated by mixing with three volumes of ice-cold ethanol and incubation at −20 °C overnight. The tRNA was pelleted by centrifugation at 10,000*g* for 60 min at 4 °C. The resulting precipitate was dried by vacuum to remove the ethanol, and the white powder was resuspended in 4 to 5 ml of a buffer containing 20 mM Tris pH 8.0 and 5 mM EDTA and stored in aliquots at −80 °C until further use.

### *In vitro* reconstitution of MnmEG reaction

All reactions were carried out in a Coy glovebox chamber (∼97% N_2_, ∼3% H_2_) under anoxic conditions. The reaction solutions such as buffers (1 M Tris–HCl pH 8.0), salt solutions (500 mM MgCl_2_ and 1 M KCl), and RNase-free water were stirred in the glovebox for a minimum of 24 h to limit the amount of oxygen in the reactions. All the reagent stock solutions such as CH_2_THF and CH_3_THF (Schircks laboratories), FAD, NADH, GTP, glycine, and DTT were prepared inside the glovebox in 50 mM Tris–HCl pH 8.0. The concentrations of these samples were determined using UV-visible spectrophotometry (Agilent 8453) using the extinction coefficient at 294 nm of 25,000 M^−1^ cm^−1^ and 32,000 M^−1^ cm^−1^ for CH_2_THF and CH_3_THF, respectively. When applicable, accurate concentrations of FAD and NADH are calculated prior to reaction assays using UV-visible absorption coefficients of 11,300 M^−1^ cm^−1^ at 450 nm and 6220 M^−1^ cm^−1^ at 340 nm, respectively. The stock solutions were dispensed into small aliquots, flash frozen, and stored at −80 °C until use.

In the reconstitution experiments, MnmE and MnmG were preincubated in 100 mM Tris–HCl pH 8.0, 100 to 150 mM KCl, and 5 % glycerol for 30 min. The typical assay mixture contained of 50 mM Tris–HCl, 5 mM MgCl_2_, 100 mM KCl, 0.5 mM exogenous unlabeled folate, 0.5 mM FAD, 0.5 mM NADH, 2 mM glycine (either unlabeled or α-^13^C labeled), 2 mM GTP, 60 to 100 ug of purified tRNA, and ∼3% to 5% glycerol per 100 μl of the reaction mixtures. The reactions were initiated by adding the preincubated MnmEG to a final concentration of 2 μM. DTT (2 mM) was included as indicated. In assays carried out with DTT-free protein preparations, the concentrations of the MnmEG were maintained at ∼10 to 20 μM. All the reaction mixtures were allowed to incubate overnight (12–14 h) at 37 °C inside the glovebox.

### Reaction clean-up, digestion, and HPLC-HRMS analysis of tRNA nucleosides

For the analysis of the tRNA nucleosides, 80 μl of the reaction was transferred to a fresh Eppendorf tube and removed from the glovebox. The tRNA was extracted from the reaction mixtures using Qiagen miRNeasy mini kit following the manufacturer's directions for the purification of RNA >18 nt as reported previously (38). RNA HiBind spin columns (Omega Biotek) were employed with Qiagen buffers. The eluted RNA from the mini columns was digested to nucleoside level using P1 nuclease (New England Biolabs), phosphodiesterase, and Fast AP (Thermo Fisher Scientific), as reported previously (38). The enzymatic digestion reaction mixtures were filtered through a 10 KDa MWCO filter prior to loading onto a Hypersil Gold C-18 column (particle size – 1.9 μM, dimensions – 2.1 mm (D) × 150 mm (L), Thermo Fisher) connected to a Vanquish UHPLC instrument (Thermo Fisher Scientific) with a photodiode array detector and a Q-Exactive mass spectrometer. The column was pre-equilibrated in buffer A containing 50 mM (NH_4_)_2_OAc pH 5.3 in Optima water at a flow rate of 0.2 ml/min. The separation of nucleosides was carried out by applying a gradient of buffer B containing 40% (v/v) Optima grade acetonitrile in water. The gradient applied is as follows: time: 0 to 3.5 min, % B: 0 to 0.8; time 3.5 to 3.75 min, % B: 0.8 to 3.2; time: 3.75 to 4.0 min, % B: 3.2 to 5.0; time: 4 to 7 min, % B:5.0 to 25.0; time: 7 to 10 min, % B: 25 to 50; time: 10 to 12 min, % B: 50 to 75; time: 12 to 12.1 min, % B: 75 to 100; time: 12.1 to 15 min, % B: 100. The eluent from the column was directed to a Q-exactive mass spectrometer, and data are recorded with an FT analyzer in positive ion mode between *m/z* 150 to 800 at a resolution of 70,000. The instrument was maintained at a capillary temperature of 320 °C, with sheath gas flow 35, auxiliary gas flow 12, and a source voltage of 3 kV. The mass corresponding to the cmnm^5^s^2^U nucleoside was included in the inclusion list, and the detector was operated in SIM (isolation range ± 2 amu) mode at a resolution of 70,000 between 3 to 7 min.

### HPLC-HRMS analysis of FAD and THF derivatives

The reaction mixtures or the concentrated protein solutions were treated with 30% w/v TCA to a final concentration of ∼5% to 10% of TCA and allowed to denature for 10 min at room temperature (RT), followed by centrifugation at 21,000*g* for 15 min to pellet out the precipitate. The supernatant was collected and injected onto a Hypersil Gold C-18 column (particle size – 1.9 μM, dimensions – 2.1 mm (D) × 150 mm (L), Thermo Fisher) connected to a Vanquish UHPLC instrument (Thermo Fisher Scientific) with a photodiode array detector and a Q-Exactive mass spectrometer. The column was pre-equilibrated in buffer A containing 0.1 % TFA in optima water at a flow rate of 0.2 ml/min, and separation was carried out by applying a gradient of buffer B containing 0.1 % TFA in optima acetonitrile. The gradient applied is as follows: time: 0 to 3 min, % B:0; time: 3 to 6 min, % B:0 to 20; time:6 to 9 min, % B:20 to 42; time: 9 to 15 min, % B: 42 to 48; time: 15 to 18 min, % B: 48 to 75; time:18 to 21 min, % B: 75 to 100. The same gradient was employed for separation and analysis of the THF analogs and FAD. Photodiode array detection is carried out between 190 to 650 nm wavelengths.

The eluent from the column was analyzed using a Q-Exactive mass spectrometer as described previously, except that the *m/z* 250 to 1300 range was used at a resolution of 140,000. The masses corresponding to FAD or R-THF molecules were included in the inclusion list, and the detector was operated in SIM mode (isolation range ± 2 amu) between 7 to 10 min and between 9 to 11 min for R-THF and FAD, respectively. A standard curve with authentic FAD was used to quantify the FAD that copurifies with MnmG using the area under 450 nm peak.

### UV-visible spectrophotometric analysis of MnmG

The UV-visible spectrophotometry analysis of intact MnmG, TCA precipitated MnmG supernatant, and authentic FAD was recorded on an Agilent 8454 instrument with a photodiode array detector under aerobic conditions between the wavelength range 190 to 800 nm. Aerobic conditions allow for oxidation of flavin cofactor for accurate quantification of the flavin. An aliquot (∼150 μM) of the MnmG solution was used, and the supernatant of the sample denatured with 30% w/v TCA was used in the analysis.

UV-visible analysis of the MnmEG complex to probe the role of a reductant is carried out on an Agilent 8453 instrument located in the Coy glovebox chamber (97.5% N_2_ and 2.5 % H_2_) under anaerobic conditions to minimize any reoxidation of the protein. An aliquot (∼200 μM) of DTT-free MnmEG was mixed with 200 μM of NADH or 1 mM DTT and allowed to incubate for 90 min at RT inside the glovebox. A blank without the reducing agent is included as well.

### Chemoenzymatic synthesis of labeled THF derivatives and concentration determination

The isotopically labeled ^13^CH_2_THF was prepared by incubating SHMT, THF (MedChem express), pyridoxal phosphate, and β-^13^C-Serine or β-CD_2_-Serine (Cambridge Isotope Laboratories). The reaction mixture generally contained ∼190 μM SHMT, ∼350 to 500 μM THF, 2 mM ^13^C labeled or deuterium labeled serine, and 1 mM pyridoxal phosphate in 100 mM Tris–HCl pH 8.0 buffer. The reactions were incubated at 37 °C for 60 min under anaerobic conditions. The reaction mixture is filtered through the 10 KDa MWCO centrifugal filter to remove the enzyme, and the resulting THF was used *in vitro* reconstitution without further purification.

In a separate preparation, after the incubation of the SHMT reaction for 60 min, the enzyme was filtered through MWCO centrifugal filter and mixed with MTHFR and NADH to prepare the isotopically labeled ^13^CH_3_THF. MTHFR and NADH were added to the filtrate to a concentration of ∼110 μM and ∼400 μM, respectively, in a total volume of 100 μl. In reactions employing NaCNBH_3_, the filtrate was mixed with 2 μl of saturated reductant (in acetonitrile) in a total volume of 100 μl. The reactions were carried out at 37 °C for 60 min under anaerobic conditions. The reaction mixtures were filtered using a 10 KDa MWCO filter, and the flow through was used in the *in vitro* reconstitution.

The composition and concentrations of species present in the RTHF produced as described before were carried out by HPLC-HRMS. Standard curves were generated using the area under the molecular ion peaks of the commercially available unlabeled CH_2_THF and CH_3_THF samples. Technical replicates were employed for each sample analyzed. Adenosine nucleoside is used as the internal control to account for the differences in ionization between samples. The molecular ion peaks *m/z* 459.17 ± 0.01 and 460.19 ± 0.01 were considered for ^13^CH_2_THF and CD_2_THF, respectively, to determine the area of isotopically labeled RTHF peaks. Similarly, the molecular ion peak *m/z* – 461.19 ± 0.01 was considered for ^13^CH_3_THF.

## Data availability

All data generated or analyzed during this study are included in this published article (and its supporting information files).

## Supporting information

This article contains supporting information (20, 33).

## Conflict of interest

The authors declare that they have no conflicts of interest with the contents of this article.
