# Supplementary material for: Elucidation of the substrate of tRNA-modifying enzymes MnmEG leads to in vitro reconstitution of an evolutionarily conserved uridine hypermodification
Source: J Biol Chem. 2022 Sep 28;298(11):102548. doi: 10.1016/j.jbc.2022.102548 (PMC9626948; doi:10.1016/j.jbc.2022.102548)
Supplement: Supplemental Fig. S1–S7 [file mmc1.docx]

**Supporting information**

**Elucidation of the substrate of tRNA-modifying enzymes MnmEG leads to *in vitro* reconstitution of an evolutionarily conserved uridine hypermodification**

Praneeth Bommisetti^1^, Anthony Young^2^, Vahe Bandarian^1^*

^1^ Department of Chemistry, University of Utah, 315 South 1400 East, Salt Lake City, UT 84112, United States

^2^Soliome Inc, 479 Jessie Street, San Francisco, CA 94103, United States

*Corresponding author: Vahe Bandarian

Email: [vahe@chem.utah.edu](mailto:vahe@chem.utah.edu).

|  | **TABLE OF CONTENTS** |  |
| --- | --- | --- |
| Figure S1 | Xm^5^U modification installation in eukaryotic mitochondrial tRNAs by MnmEG homologs | Page S3 |
| Figure S2 | Structures of tetrahydrofolic acid and its derivatives | Page S4 |
| Figure S3 | Mnm5s2U modification dependency on various folate enzymes | Page S5 |
| Figure S4 | PAGE analysis of purified proteins and tRNA substrate | Page S6 |
| Figure S5 | Analysis of small molecules that copurify with MnmE | Page S7 |
| Figure S6 | HPLC-HRMS analysis of the tRNA nucleosides with CH_3_THF as the exogenous input folate. | Page S8 |
| Figure S7 | Reduction of free flavin in solution by DTT | Page S9 |
|  | References | Page S9 |

| **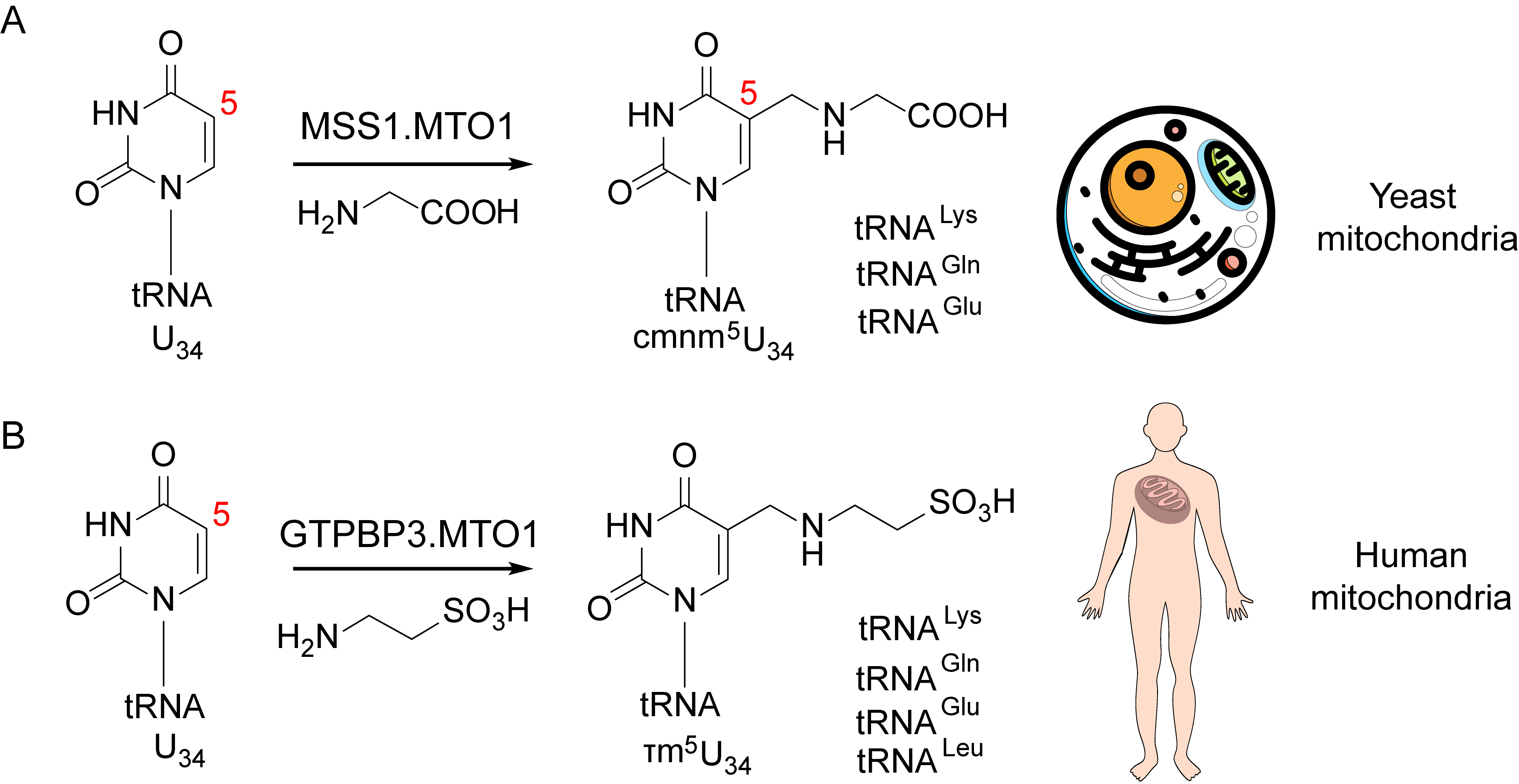** |
| --- |
| **Figure S1. xm^5^U modification installation in eukaryotic mitochondrial tRNAs by MnmEG homologs. A.** The cmnm^5^ pathway in the yeast mitochondria. **B**. The τm^5^ pathway in human mitochondria. |

| **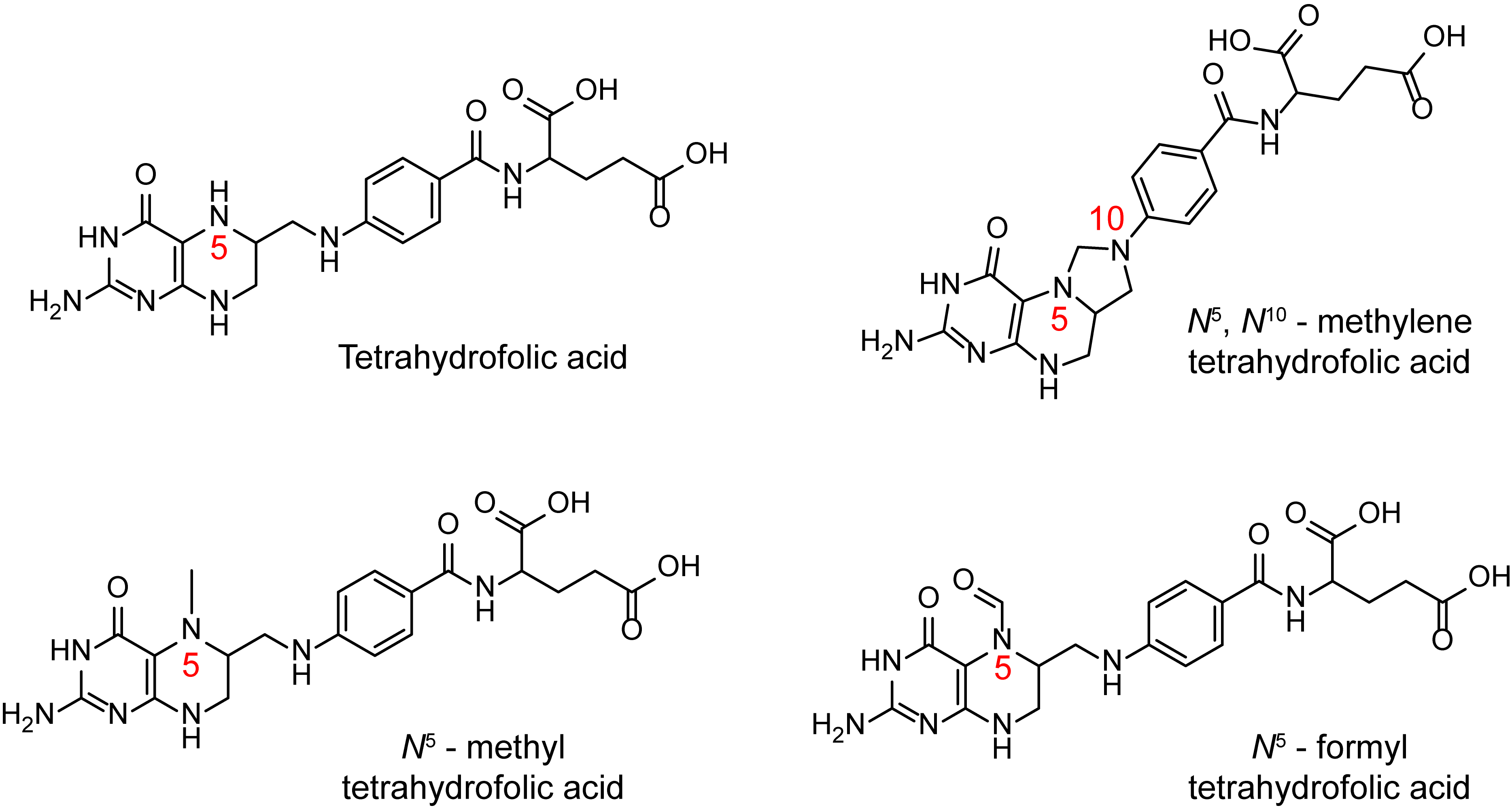** |
| --- |
| **Figure S2. Structures of tetrahydrofolic acid and its derivatives.** Position 5 (or 10) on these molecules is labeled red. |

| **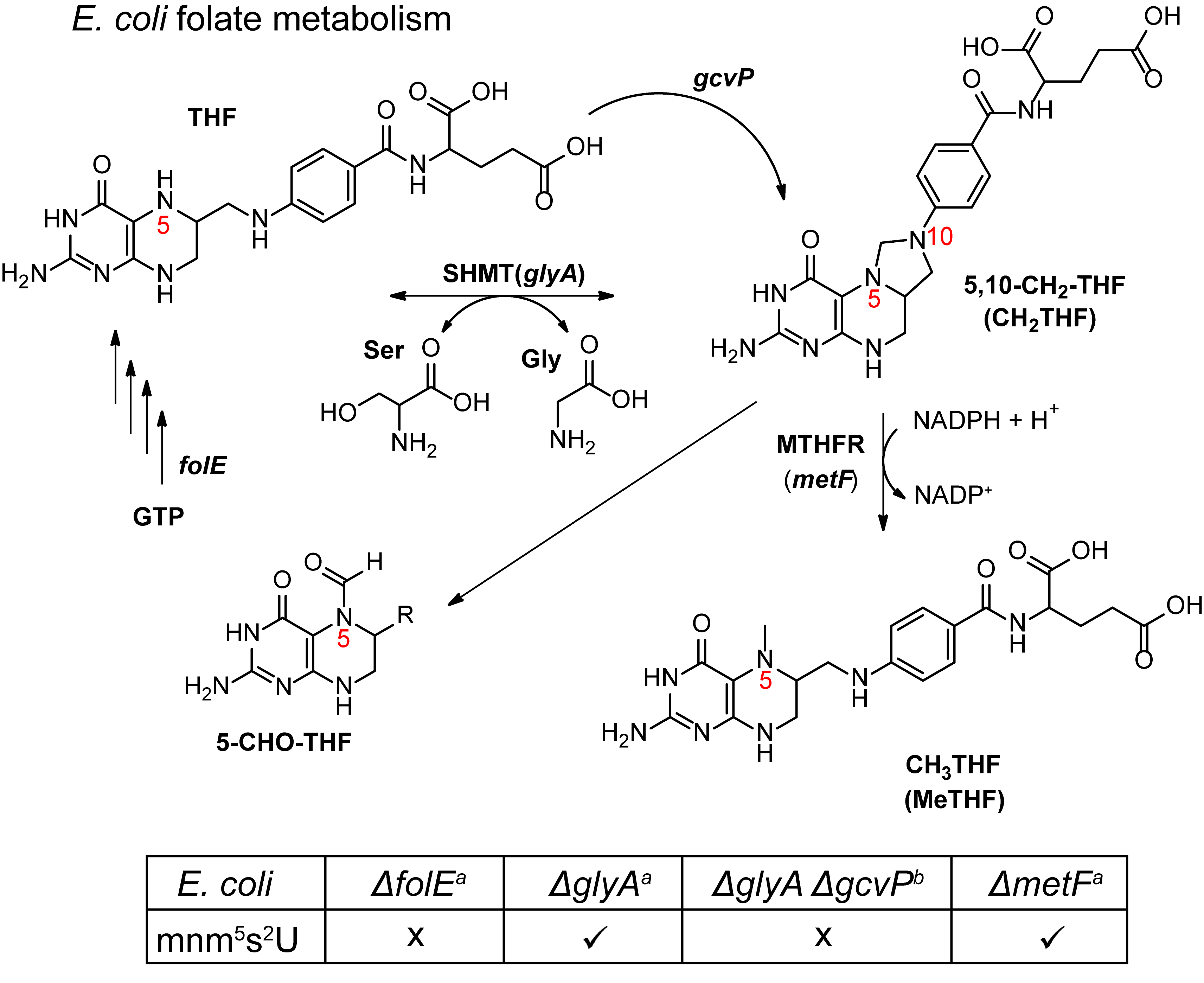** |
| --- |
| **Figure S3. Mnm^5^s^2^U modification dependency on various folate enzymes.** The data depicted in the table is an interpretation of the *in vivo* studies in previous reports (1, 2). |

| **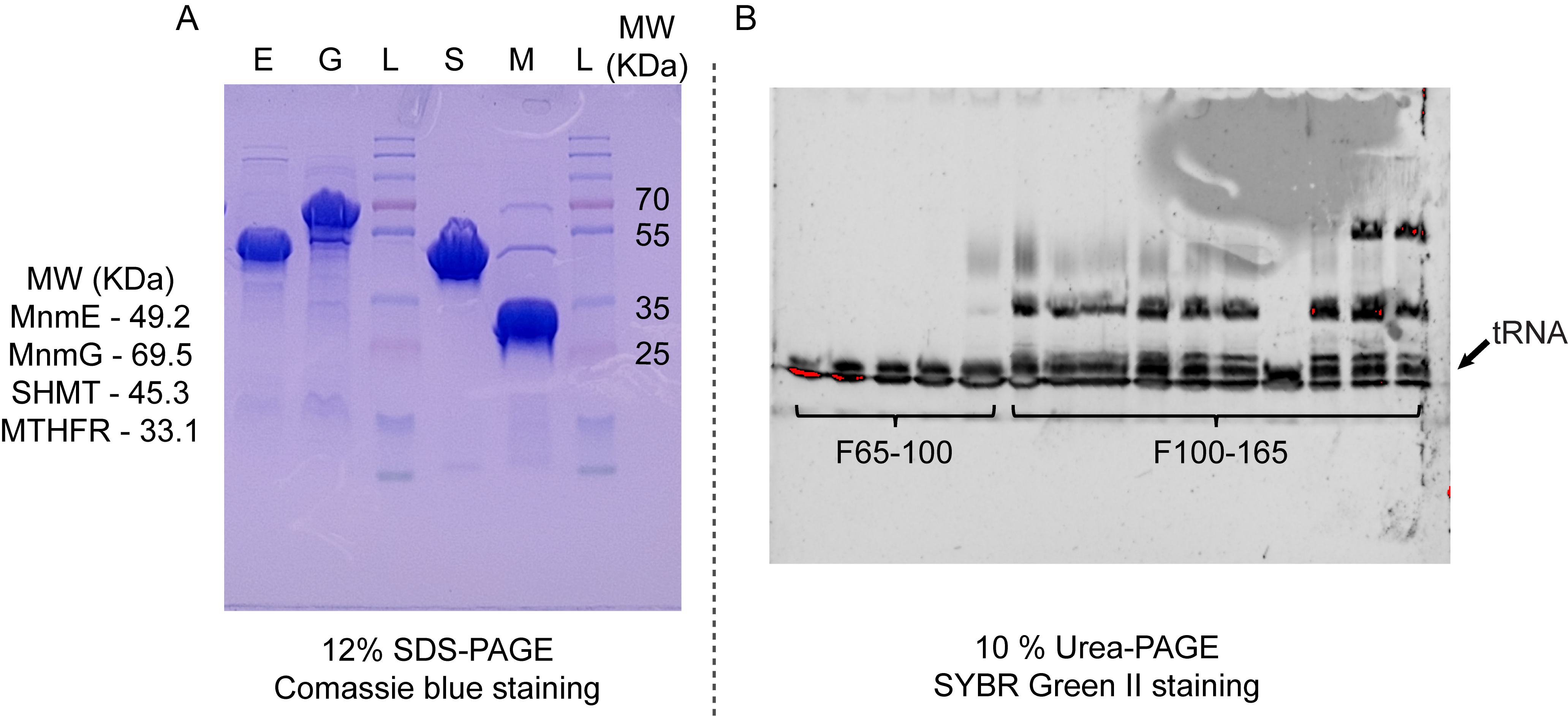** |
| --- |
| **Figure S4. PAGE analysis of purified proteins and tRNA substrate. A.** Representative SDS-PAGE analysis of the purified MnmE, MnmG, SHMT, and MTHFR proteins. The corresponding lanes are labeled as follows. E: MnmE, G: MnmG, S: SHMT, M: MTHFR, L: Ladder, MW: molecular weight. The molecular weight of the proteins is listed on the left-hand side of the A. **B.** Representative urea-PAGE analysis of tRNA fractions from DEAE column FPLC purification. The fractions F65-100 and F100-165 were pooled separately, although both pools contain s^2^U. Therefore, tRNA from both fraction pools were mixed and employed in the reaction. |

| 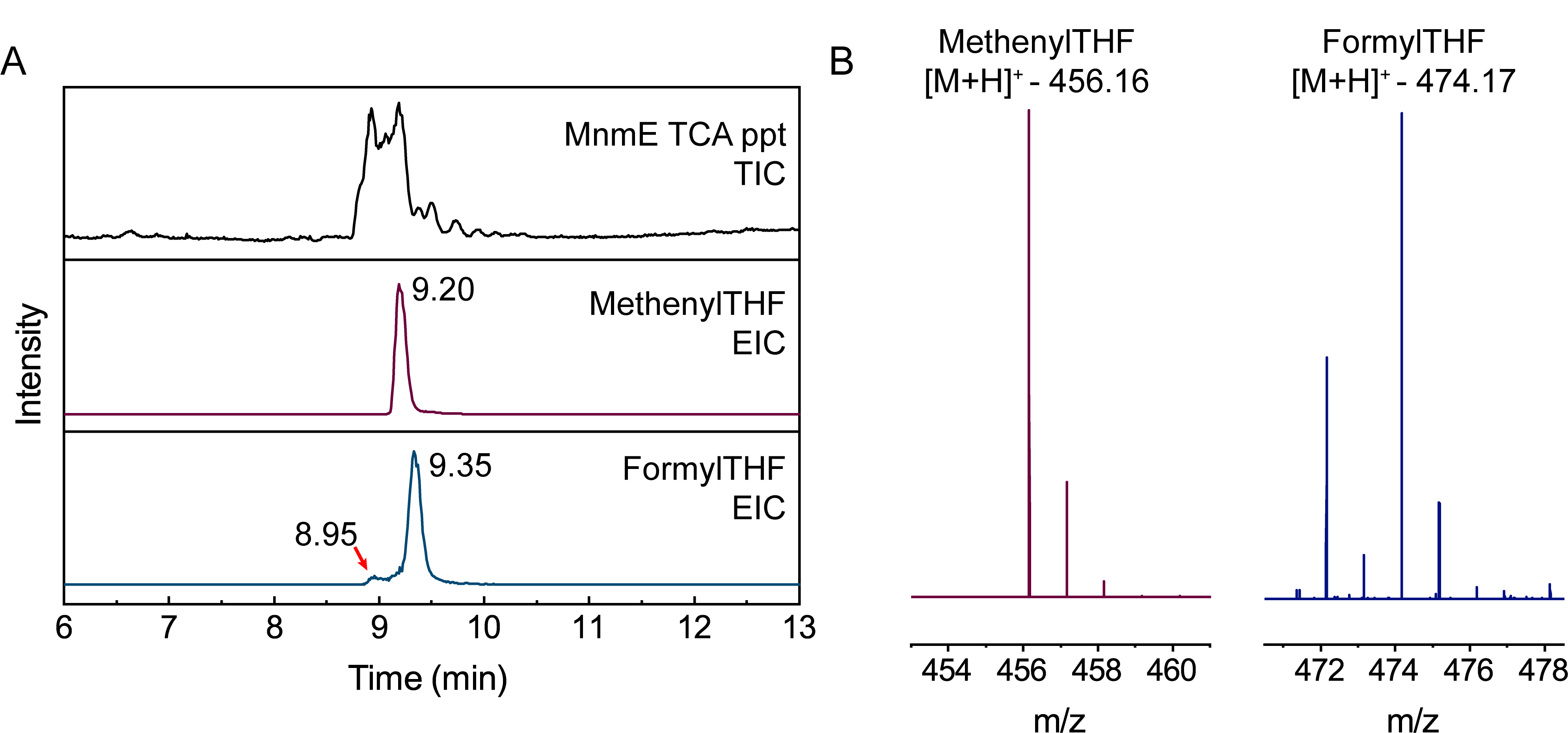 |
| --- |
| **Figure S5. Analysis of small molecules that copurify with MnmE. A.** The TIC of MnmE TCA precipitate (black) and EICs of methenylTHF (m/z – 456.16±0.01, red), and formylTHF (m/z - 474.17±0.01, green) from MnmE supernatant. The retention times are labeled. The black TIC trace depicted is the same as the one depicted in Figure 2A but has been included here for comparison. **B.** Representative mass spectra of the methenylTHF and formylTHF observed in the MnmE supernatant sample |

| **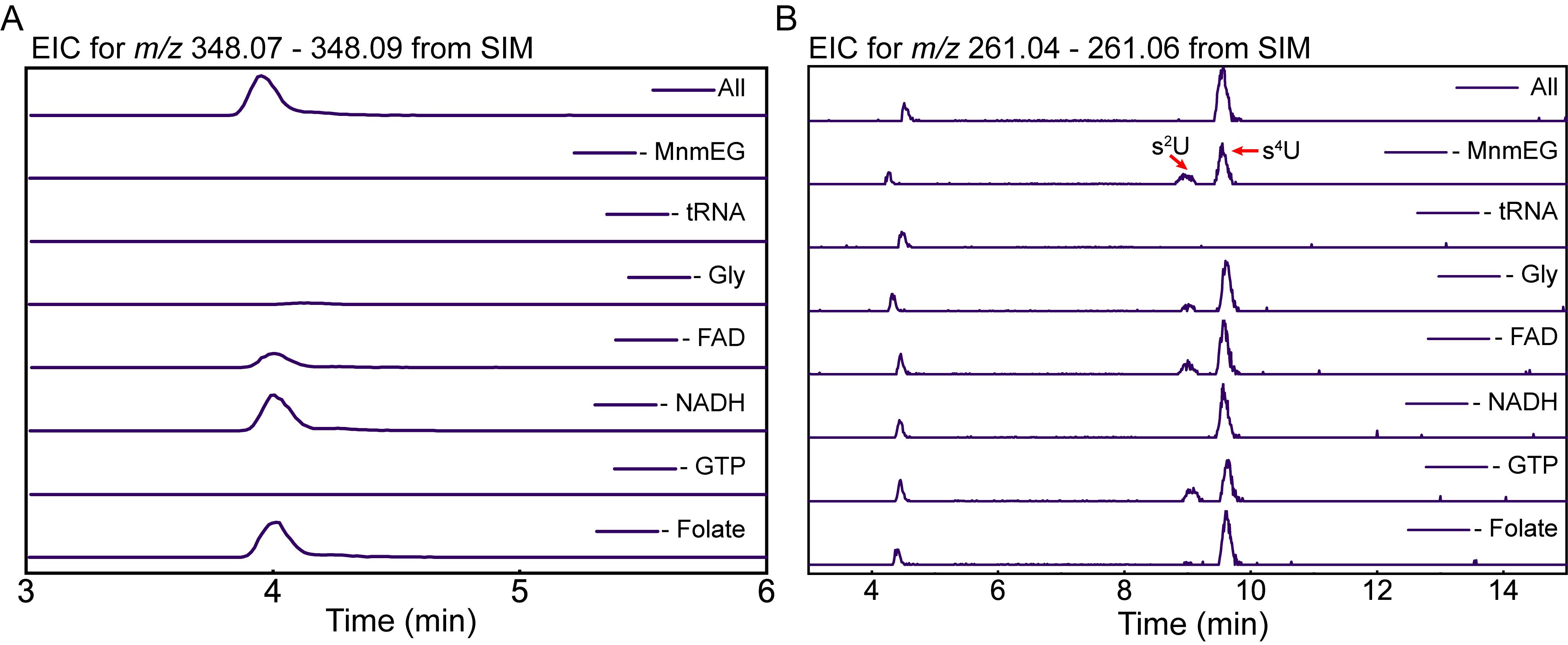** |
| --- |
| **Figure S6. HPLC-HRMS analysis of the tRNA nucleosides with CH_3_THF as the input exogenous folate. A**. Representative EICs of the cmnm^5^s^2^U (*m/z* – 348.08±0.01) product from reaction samples obtained by omitting one reagent at a time. **B**. EICs of the s^2^U (*m/z* - 261.05±0.01) nucleoside from reaction samples obtained by omitting one reagent at a time. The "All" trace in A and B are the same as the "+MeTHF" traces from **Figure 3C and 3D** and are replicated here for comparison. The omitted reagents are labelled for each of the traces in A and B |

| **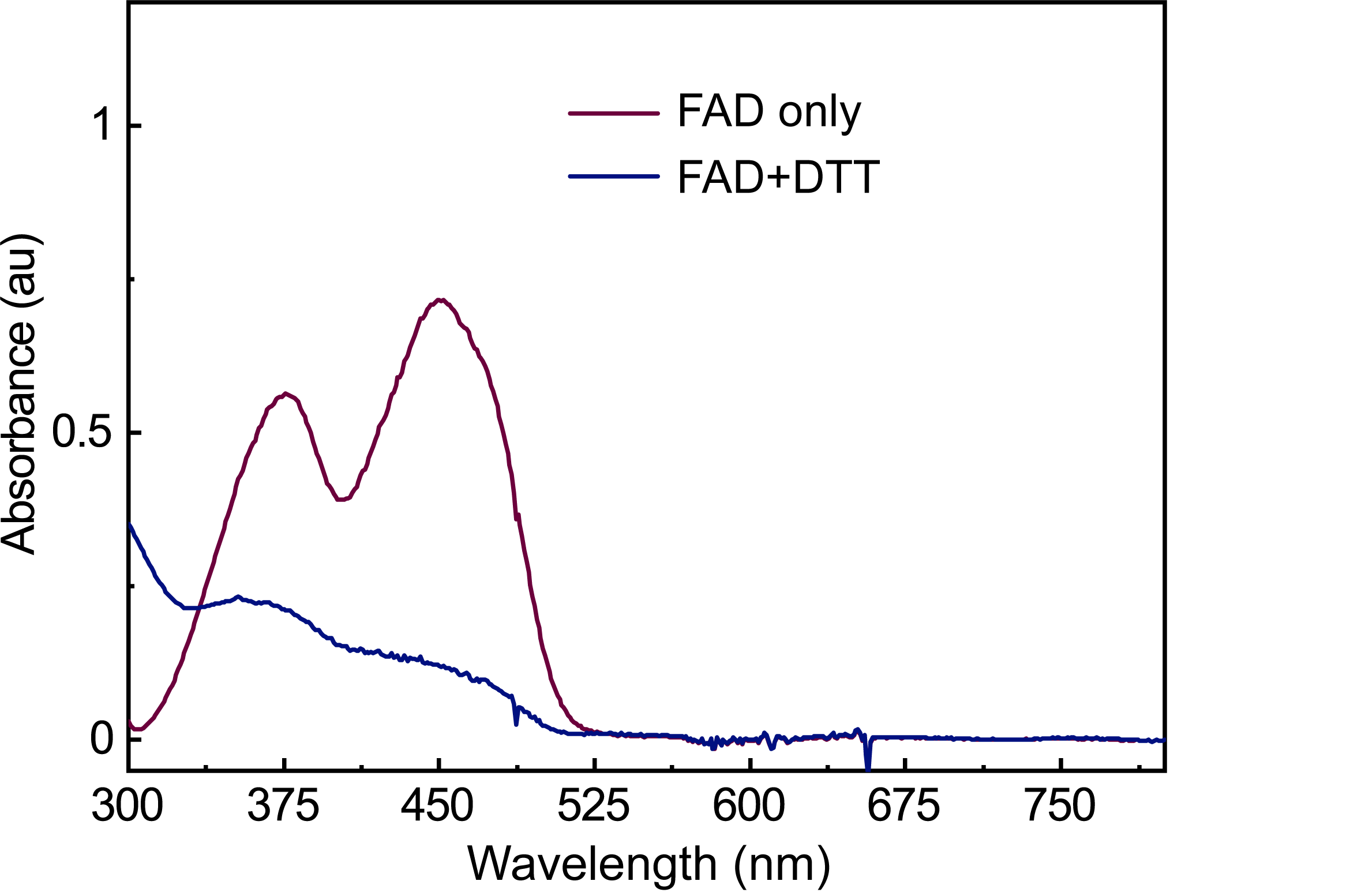** |
| --- |
| **Figure S7. Reduction of free flavin in solution by DTT.** UV-vis absorbance spectrum depicting the loss of characteristic flavin spectrum upon incubation with DTT. Approximately 50 µM of FAD was incubated with 2 mM DTT overnight in deionized water. |

References

1. Moukadiri, I., Prado, S., Piera, J., Velázquez-campoy, A., Björk, G. R., and Armengod, M. E. (2009) Evolutionarily conserved proteins MnmE and GidA catalyze the formation of two methyluridine derivatives at tRNA wobble positions. *Nucleic Acids Res.* **37**, 7177–7193

2. Waller, J. C., Alvarez, S., Naponelli, V., Lara-Nuñez, A., Blaby, I. K., Da Silva, V., Ziemak, M. J., Vickers, T. J., Beverley, S. M., Edison, A. S., Rocca, J. R., Gregory, J. F., De Crécy-Lagard, V., and Hanson, A. D. (2010) A role for tetrahydrofolates in the metabolism of iron-sulfur clusters in all domains of life. *Proc. Natl. Acad. Sci. U. S. A.* **107**, 10412–10417
